# (1*R*,3*S*)-1,1′-(1,3-Dihydro-2-benzofuran-1,3-diyl)bis(1,3-dimethyl­urea)

**DOI:** 10.1107/S1600536808040828

**Published:** 2008-12-06

**Authors:** Bushra Maliha, Muhammad Ilyas Tariq, M. Nawaz Tahir, Ishtiaq Hussain, Hamid Latif Siddiqui

**Affiliations:** a Institute of Chemistry, University of the Punjab, Lahore-54590, Pakistan; b Department of Chemistry, University of Sargodha, Sargodha, Pakistan; c Department of Physics, University of Sargodha, Sargodha, Pakistan

## Abstract

In the mol­ecule of the title compound, C_14_H_20_N_4_O_3_, the five-membered ring adopts an envelope conformation with the O atom displaced by 0.207 (3) Å from the plane of the other ring atoms. Intra­molecular C—H⋯O hydrogen bonds result in the formation of three five-membered rings having envelope conformations. In the crystal structure, inter­molecular N—H⋯O hydrogen bonds link the mol­ecules, forming *R*_2_^2^(20) ring motifs, which produce two-dimensional polymeric sheets extending along the *b* axis. There are also two C—H⋯π inter­actions. The H atoms of one of the methyl groups are disordered over two positions and were refined with occupancies of 0.50.

## Related literature

For general background, see: Veeraraghavan *et al.* (1996 ▶); Lin *et al.* (2005 ▶); Roy & Sarkar (2005 ▶); Harper *et al.* (2003 ▶); Tsi & Tan (1997 ▶). For related structures, see: Maliha *et al.* (2007 ▶, 2009 ▶); Maliha, Hussain *et al.* (2008 ▶); Maliha, Tariq *et al.* (2008 ▶). For ring-motifs, see: Bernstein *et al.* (1995 ▶).For bond lengths and angles in 3-[(2-hydr­oxy-5-nitro­phen­yl)amino]-2-benzofuran-1(3*H*)-one monohydrate, see: Odabaşoğlu & Büyükgüngör (2006 ▶).
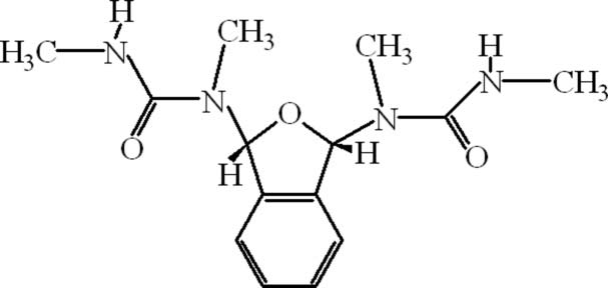


## Experimental

### 

#### Crystal data

C_14_H_20_N_4_O_3_
*M* *_r_* = 292.34
Orthorhombic, 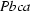

*a* = 14.6322 (6) Å
*b* = 9.1014 (3) Å
*c* = 21.2307 (9) Å
*V* = 2827.37 (19) Å^3^
*Z* = 8
Mo *K*α radiation
μ = 0.10 mm^−1^
*T* = 296 (2) K
0.30 × 0.10 × 0.06 mm

#### Data collection

Bruker Kappa APEXII CCD diffractometer
Absorption correction: multi-scan (*SADABS*; Bruker, 2005 ▶) *T*_min_ = 0.982, *T*_max_ = 0.989
19271 measured reflections
3257 independent reflections
2741 reflections with *I* > 2σ(*I*)
*R*_int_ = 0.033

#### Refinement

*R*[*F* ^2^ > 2σ(*F* ^2^)] = 0.038
*wR*(*F* ^2^) = 0.150
*S* = 1.02
3257 reflections
206 parameters
H atoms treated by a mixture of independent and constrained refinement
Δρ_max_ = 0.32 e Å^−3^
Δρ_min_ = −0.30 e Å^−3^

### 

Data collection: *APEX2* (Bruker, 2007 ▶); cell refinement: *SAINT* (Bruker, 2007 ▶); data reduction: *SAINT*; program(s) used to solve structure: *SHELXS97* (Sheldrick, 2008 ▶); program(s) used to refine structure: *SHELXL97* (Sheldrick, 2008 ▶); molecular graphics: *ORTEP-3 for Windows* (Farrugia, 1997 ▶) and *PLATON* (Spek, 2003 ▶); software used to prepare material for publication: *WinGX* (Farrugia, 1999 ▶) and *PLATON*.

## Supplementary Material

Crystal structure: contains datablocks global, I. DOI: 10.1107/S1600536808040828/hk2591sup1.cif

Structure factors: contains datablocks I. DOI: 10.1107/S1600536808040828/hk2591Isup2.hkl

Additional supplementary materials:  crystallographic information; 3D view; checkCIF report

## Figures and Tables

**Table 1:** Hydrogen-bond geometry (Å, °)

*D*—H⋯*A*	*D*—H	H⋯*A*	*D*⋯*A*	*D*—H⋯*A*
N2—H2*N*⋯O3^i^	0.806 (19)	2.062 (19)	2.8229 (16)	157.4 (19)
N4—H4*N*⋯O2^i^	0.858 (18)	2.006 (18)	2.8322 (15)	161.2 (18)
C1—H1⋯O3	0.987 (17)	2.263 (17)	2.7205 (16)	107.0 (12)
C8—H8⋯O2	1.003 (17)	2.239 (17)	2.7505 (17)	110.1 (12)
C11—H11*A*⋯O2	0.96	2.39	2.7730 (18)	103.0
C9—H9*B*⋯CgA	0.96	2.6600	3.0207 (14)	103.0
C12—H12*B*⋯CgA	0.96	2.7200	3.0046 (15)	98.0

Symmetry code: (i) 
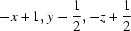
. *CgA* is the centroid of the O1/C1/C2/C7/C8 ring.

**Fig. 1.**
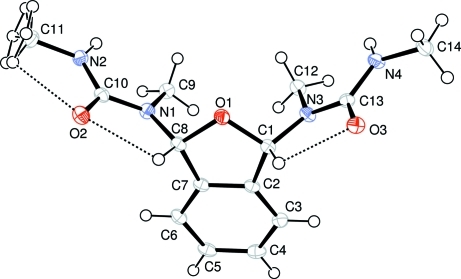
The molecular structure of the title molecule, with the atom-numbering scheme. Displacement ellipsoids are drawn at the 50% probability level. Hydrogen bonds are shown as dashed lines.

**Fig. 2.**
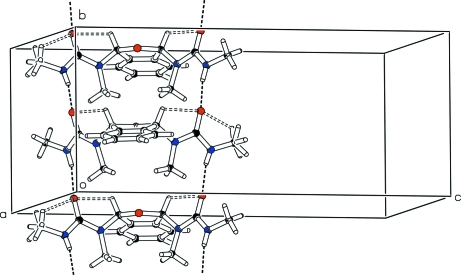
A partial packing diagram of the title compound. Hydrogen bonds are shown as dashed lines.

## supplementary crystallographic information

### Comment

Isobenzofurans exhibit anticonvulsant, antitumour and antiasthamatic properties (Veeraraghavan *et al.*, 1996). These compounds have several biological activities, such as antioxidant, antimycotic, cytotoxic, antimicrobial, herbicidal, analgesic and pesticidal activities (Lin *et al.*, 2005, Roy & Sarkar, 2005, Harper *et al.*, 2003). These are known to exhibit hypotensive and vasorelaxant properties (Tsi & Tan, 1997). We report herein the synthesis and crystal structure of the title compound. This study is in countinuation to the formation of derivatives of *O*-phthaldehyde with different ureas (Maliha *et al.*, 2007; Maliha, Hussain *et al.*, 2008; Maliha, Tariq *et al.*, 2008).

The molecule of the title compound is essentially symmetric about the mirror plane passing through the O1 atom of the 2-benzofuran ring system as far as the chemical structure is concerned. But, the intramolecular C-H···O hydrogen bonds (Table 1) disturb this symmetry. Due to this reason, there exist R and S-configurations at C1 and C8 atoms, respectively. The bond legths and angles in the 2-benzofuran ring system are in accordance with the corresponding values in 3-[(2-Hydroxy-5-nitrophenyl)amino]-2-benzofuran-1(3*H*)-one monohydrate (Odabaşoĝlu & Büyükgüngör, 2006). Ring B (C2-C7) is, of course, planar, while the five-membered ring A (O1/C1/C2/C7/C8) adopts envelope conformation with O1 atom displaced by -0.207 (3) Å from the plane of the other ring atoms. The intramolecular C-H···O hydrogen bonds (Table 1) result in the formation of three five-membered rings: C (O3/N3/C1/C13/H1), D (O2/N1/C8/C10/H8) and E (O2/N2/C10/C11/H11A), having envelope conformations with N3, N1 and H11A atoms displaced by 0.191 (3), -0.155 (3) and -0.265 (3) Å from the planes of the other rings atoms, respectively.

In the crystal structure, intermolecular N-H···O hydrogen bonds (Table 1) link the molecules to form *R*_2_^2^(20) ring motifs (Bernstein *et al.*, 1995), which are joint in such a fashion that the 2-benzofuran rings are in cis and trans positions. They produce two dimensional polymeric sheets extending along the b axis (Fig 2). There also exist two C–H···π interactions (Table 1).

### Experimental

For the preparation of the title compound, *O*-phthaldehyde (200 mmol), *N*,*N*-dimethylurea (200 mmol) and a few drops of HCl were refluxed in ethanol. Colorless precipitate obtained was crystallized in the solution of methanol:acetone (9:1) by slow evaporation at room temperature.

### Refinement

The hydrogen atoms of the C11 methyl group were disordered over two positions. During the refinement process the disordered atoms were refined with occupancies of 0.50. H1, H8 (for CH) and H2N, H4N (for NH) atoms were located in difference syntheses and refined [C-H = 0.987 (17) and 1.003 (17) Å, U_iso_(H) = 1.2U_eq_(C); N-H = 0.806 (19) and 0.858 (18) Å, U_iso_(H) = 1.5U_eq_(N). The remaining H atoms were positioned geometrically, with C-H = 0.93 and 0.96 Å for aromatic and methyl H, respectively, and constrained to ride on their parent atoms with U_iso_(H) = xU_eq_(C), where x = 1.5 for methyl H and x = 1.2 for aromatic H atoms.

### Crystal data

**Table:**

C_14_H_20_N_4_O_3_	*F*(000) = 1008
*M**_r_* = 292.34	*D*_x_ = 1.374 Mg m^−^^3^
Orthorhombic, *P**b**c**a*	Mo *K*α radiation, λ = 0.71073 Å
Hall symbol: -P 2ac 2ab	Cell parameters from 2741 reflections
*a* = 14.6322 (6) Å	θ = 1.9–27.6°
*b* = 9.1014 (3) Å	µ = 0.10 mm^−^^1^
*c* = 21.2307 (9) Å	*T* = 296 K
*V* = 2827.37 (19) Å^3^	Needle, colorless
*Z* = 8	0.30 × 0.10 × 0.06 mm

### Data collection

**Table:**

Bruker Kappa APEXII CCD diffractometer	3257 independent reflections
Radiation source: fine-focus sealed tube	2741 reflections with *I* > 2σ(*I*)
graphite	*R*_int_ = 0.033
Detector resolution: 7.50 pixels mm^-1^	θ_max_ = 27.6°, θ_min_ = 1.9°
ω scans	*h* = −18→19
Absorption correction: multi-scan (*SADABS*; Bruker, 2005)	*k* = −11→11
*T*_min_ = 0.982, *T*_max_ = 0.989	*l* = −27→27
19271 measured reflections	

### Refinement

**Table:**

Refinement on *F*^2^	Primary atom site location: structure-invariant direct methods
Least-squares matrix: full	Secondary atom site location: difference Fourier map
*R*[*F*^2^ > 2σ(*F*^2^)] = 0.038	Hydrogen site location: inferred from neighbouring sites
*wR*(*F*^2^) = 0.150	H atoms treated by a mixture of independent and constrained refinement
*S* = 1.02	*w* = 1/[σ^2^(*F*_o_^2^) + (0.1147*P*)^2^ + 0.2587*P*] where *P* = (*F*_o_^2^ + 2*F*_c_^2^)/3
3257 reflections	(Δ/σ)_max_ = 0.001
206 parameters	Δρ_max_ = 0.32 e Å^−^^3^
0 restraints	Δρ_min_ = −0.30 e Å^−^^3^

### Special details

**Table:**

Geometry. Bond distances, angles *etc*. have been calculated using the rounded fractional coordinates. All su's are estimated from the variances of the (full) variance-covariance matrix. The cell e.s.d.'s are taken into account in the estimation of distances, angles and torsion angles
Refinement. Refinement of *F*^2^ against ALL reflections. The weighted *R*-factor *wR* and goodness of fit *S* are based on *F*^2^, conventional *R*-factors *R* are based on *F*, with *F* set to zero for negative *F*^2^. The threshold expression of *F*^2^ > σ(*F*^2^) is used only for calculating *R*-factors(gt) *etc*. and is not relevant to the choice of reflections for refinement. *R*-factors based on *F*^2^ are statistically about twice as large as those based on *F*, and *R*- factors based on ALL data will be even larger.

### Fractional atomic coordinates and isotropic or equivalent isotropic displacement parameters (Å^2^)

**Table:**

	*x*	*y*	*z*	*U*_iso_*/*U*_eq_	Occ. (<1)
O1	0.41467 (6)	0.45600 (11)	0.23235 (4)	0.0174 (3)	
O2	0.34799 (7)	0.54659 (10)	0.39413 (5)	0.0198 (3)	
O3	0.51470 (7)	0.55115 (10)	0.08040 (4)	0.0182 (3)	
N1	0.34567 (8)	0.35646 (12)	0.32370 (5)	0.0172 (3)	
N2	0.38830 (8)	0.31870 (14)	0.42697 (5)	0.0192 (3)	
N3	0.45143 (8)	0.35663 (12)	0.13270 (5)	0.0166 (3)	
N4	0.58841 (8)	0.33324 (13)	0.07804 (6)	0.0184 (3)	
C1	0.39065 (9)	0.45278 (15)	0.16603 (6)	0.0158 (4)	
C2	0.29157 (9)	0.40893 (14)	0.16576 (6)	0.0156 (4)	
C3	0.23533 (9)	0.37551 (15)	0.11520 (7)	0.0190 (4)	
C4	0.14426 (10)	0.34209 (16)	0.12735 (7)	0.0216 (4)	
C5	0.11093 (9)	0.34332 (15)	0.18873 (7)	0.0202 (4)	
C6	0.16762 (9)	0.37606 (15)	0.23927 (7)	0.0183 (4)	
C7	0.25847 (9)	0.40992 (15)	0.22654 (6)	0.0157 (4)	
C8	0.33352 (9)	0.45371 (15)	0.27125 (6)	0.0161 (3)	
C9	0.35403 (9)	0.19953 (15)	0.31148 (6)	0.0180 (4)	
C10	0.36122 (9)	0.41503 (15)	0.38270 (6)	0.0163 (4)	
C11	0.40137 (10)	0.36344 (17)	0.49181 (7)	0.0229 (4)	
C12	0.45527 (10)	0.20371 (14)	0.15296 (7)	0.0187 (4)	
C13	0.51929 (9)	0.42070 (14)	0.09633 (6)	0.0151 (3)	
C14	0.65525 (9)	0.38690 (17)	0.03293 (7)	0.0216 (4)	
H1	0.3955 (12)	0.5527 (19)	0.1483 (8)	0.0190*	
H2N	0.4057 (14)	0.238 (2)	0.4170 (9)	0.0287*	
H3	0.25789	0.37544	0.07425	0.0228*	
H4	0.10534	0.31875	0.09419	0.0259*	
H4N	0.5954 (13)	0.244 (2)	0.0902 (9)	0.0276*	
H5	0.04970	0.32187	0.19602	0.0243*	
H6	0.14554	0.37537	0.28035	0.0220*	
H8	0.3231 (12)	0.5534 (18)	0.2901 (8)	0.0193*	
H9A	0.32072	0.14578	0.34289	0.0269*	
H9B	0.32957	0.17763	0.27058	0.0269*	
H9C	0.41729	0.17176	0.31291	0.0269*	
H11A	0.37241	0.45675	0.49861	0.0344*	0.500
H11B	0.37481	0.29147	0.51934	0.0344*	0.500
H11C	0.46556	0.37176	0.50047	0.0344*	0.500
H11D	0.43611	0.28991	0.51367	0.0344*	0.500
H11E	0.43371	0.45518	0.49294	0.0344*	0.500
H11F	0.34296	0.37489	0.51181	0.0344*	0.500
H12A	0.49792	0.19423	0.18707	0.0280*	
H12B	0.39578	0.17296	0.16673	0.0280*	
H12C	0.47463	0.14323	0.11840	0.0280*	
H14A	0.68780	0.46846	0.05079	0.0324*	
H14B	0.69754	0.30969	0.02295	0.0324*	
H14C	0.62456	0.41798	−0.00475	0.0324*	

### Atomic displacement parameters (Å^2^)

**Table:**

	*U*^11^	*U*^22^	*U*^33^	*U*^12^	*U*^13^	*U*^23^
O1	0.0130 (5)	0.0212 (5)	0.0180 (5)	−0.0019 (3)	0.0006 (3)	−0.0010 (4)
O2	0.0210 (5)	0.0152 (5)	0.0231 (5)	−0.0014 (4)	0.0019 (4)	−0.0037 (4)
O3	0.0177 (5)	0.0155 (5)	0.0214 (5)	−0.0014 (3)	0.0001 (4)	0.0022 (4)
N1	0.0202 (6)	0.0135 (6)	0.0179 (6)	0.0002 (4)	−0.0003 (4)	−0.0011 (4)
N2	0.0206 (6)	0.0189 (6)	0.0180 (6)	0.0029 (4)	−0.0016 (5)	−0.0031 (5)
N3	0.0153 (6)	0.0130 (5)	0.0216 (6)	0.0005 (4)	0.0032 (4)	0.0012 (4)
N4	0.0149 (6)	0.0172 (6)	0.0232 (6)	0.0019 (4)	0.0025 (4)	0.0031 (5)
C1	0.0149 (6)	0.0149 (7)	0.0176 (6)	0.0004 (5)	0.0007 (5)	−0.0003 (5)
C2	0.0144 (6)	0.0121 (6)	0.0204 (7)	0.0015 (5)	0.0001 (5)	0.0008 (5)
C3	0.0189 (7)	0.0185 (7)	0.0195 (6)	0.0014 (5)	−0.0011 (5)	0.0009 (5)
C4	0.0184 (7)	0.0186 (7)	0.0278 (8)	0.0001 (5)	−0.0063 (5)	−0.0010 (6)
C5	0.0131 (6)	0.0157 (7)	0.0318 (8)	−0.0006 (5)	−0.0009 (5)	−0.0009 (5)
C6	0.0167 (7)	0.0145 (6)	0.0238 (7)	0.0002 (5)	0.0037 (5)	0.0001 (5)
C7	0.0151 (6)	0.0111 (6)	0.0209 (7)	0.0009 (5)	0.0002 (5)	0.0001 (5)
C8	0.0136 (6)	0.0154 (6)	0.0193 (6)	−0.0005 (5)	0.0013 (5)	0.0000 (5)
C9	0.0201 (7)	0.0138 (7)	0.0200 (6)	−0.0014 (5)	−0.0013 (5)	−0.0008 (5)
C10	0.0115 (6)	0.0174 (7)	0.0199 (6)	−0.0020 (5)	0.0020 (5)	−0.0017 (5)
C11	0.0233 (7)	0.0261 (8)	0.0194 (7)	−0.0018 (6)	−0.0034 (5)	−0.0018 (5)
C12	0.0198 (7)	0.0138 (7)	0.0224 (6)	0.0007 (5)	0.0031 (5)	0.0016 (5)
C13	0.0136 (6)	0.0154 (6)	0.0162 (6)	−0.0012 (5)	−0.0020 (5)	−0.0003 (5)
C14	0.0157 (7)	0.0251 (8)	0.0240 (7)	−0.0003 (5)	0.0036 (5)	0.0006 (6)

### Geometric parameters (Å, °)

**Table:**

O1—C1	1.4515 (15)	C7—C8	1.5053 (18)
O1—C8	1.4465 (16)	C1—H1	0.987 (17)
O2—C10	1.2370 (16)	C3—H3	0.9300
O3—C13	1.2363 (16)	C4—H4	0.9300
N1—C8	1.4335 (17)	C5—H5	0.9300
N1—C9	1.4568 (17)	C6—H6	0.9300
N1—C10	1.3802 (17)	C8—H8	1.003 (17)
N2—C10	1.3450 (18)	C9—H9A	0.9600
N2—C11	1.4482 (18)	C9—H9B	0.9600
N3—C1	1.4344 (17)	C9—H9C	0.9600
N3—C12	1.4578 (17)	C11—H11A	0.9600
N3—C13	1.3864 (17)	C11—H11B	0.9600
N4—C13	1.3444 (18)	C11—H11C	0.9600
N4—C14	1.4534 (19)	C11—H11D	0.9600
N2—H2N	0.806 (19)	C11—H11E	0.9600
N4—H4N	0.858 (18)	C11—H11F	0.9600
C1—C2	1.5037 (19)	C12—H12A	0.9600
C2—C7	1.3783 (18)	C12—H12B	0.9600
C2—C3	1.3863 (19)	C12—H12C	0.9600
C3—C4	1.391 (2)	C14—H14A	0.9600
C4—C5	1.392 (2)	C14—H14B	0.9600
C5—C6	1.389 (2)	C14—H14C	0.9600
C6—C7	1.3911 (19)		
			
O2···C12^i^	3.3662 (18)	H1···C4^iv^	2.734 (17)
O2···N4^i^	2.8322 (15)	H1···C5^iv^	2.782 (17)
O3···N2^i^	2.8229 (16)	H2N···C9	2.390 (19)
O3···C9^i^	3.2836 (16)	H2N···H9A	2.1700
O1···H5^ii^	2.7800	H2N···H9C	2.3000
O1···H12A	2.8400	H2N···O3^vi^	2.062 (19)
O2···H11A	2.3900	H3···H11B^vii^	2.5700
O2···H11E	2.5800	H4N···C12	2.473 (19)
O2···H8	2.239 (17)	H4N···H12A	2.5400
O2···H14A^iii^	2.7100	H4N···H12C	2.0800
O2···H9A^iv^	2.8400	H4N···O2^vi^	2.006 (18)
O2···H4N^i^	2.006 (18)	H5···O1^iii^	2.7800
O2···H12C^i^	2.7500	H5···C9^iii^	3.0800
O3···H14C	2.7100	H5···H9C^iii^	2.3800
O3···H1	2.263 (17)	H6···C9^iv^	3.0200
O3···H14A	2.7200	H8···O2	2.239 (17)
O3···H2N^i^	2.062 (19)	H8···C9^iv^	2.948 (17)
O3···H9C^i^	2.7100	H8···H9A^iv^	2.5300
O3···H14C^v^	2.6100	H8···H9B^iv^	2.5400
N2···O3^vi^	2.8229 (16)	H9A···N2	2.5800
N4···O2^vi^	2.8322 (15)	H9A···H2N	2.1700
N2···H9A	2.5800	H9A···O2^viii^	2.8400
N2···H9C	2.8000	H9A···H8^viii^	2.5300
N3···H11D^vii^	2.8700	H9B···C6	3.0500
N4···H12C	2.5500	H9B···C7	2.5400
N4···H11D^vii^	2.8400	H9B···H12B	2.4100
C1···C5^iv^	3.5871 (19)	H9B···C6^viii^	2.8200
C5···C12^iv^	3.5034 (19)	H9B···C7^viii^	2.9100
C5···C1^viii^	3.5871 (19)	H9B···H8^viii^	2.5400
C6···C9^iv^	3.3344 (19)	H9C···N2	2.8000
C6···C9	3.5173 (19)	H9C···H2N	2.3000
C7···C9^iv^	3.5930 (19)	H9C···O3^vi^	2.7100
C9···C7^viii^	3.5930 (19)	H9C···H5^ii^	2.3800
C9···O3^vi^	3.2836 (16)	H11A···O2	2.3900
C9···C6^viii^	3.3344 (19)	H11B···C12^ix^	3.0700
C9···C6	3.5173 (19)	H11B···H3^ix^	2.5700
C10···C14^iii^	3.5153 (19)	H11C···H12C^ix^	2.5100
C11···C12^ix^	3.564 (2)	H11D···N3^ix^	2.8700
C12···O2^vi^	3.3662 (18)	H11D···N4^ix^	2.8400
C12···C11^vii^	3.564 (2)	H11D···C12^ix^	2.9700
C12···C5^viii^	3.5034 (19)	H11D···C13^ix^	2.8700
C14···C10^ii^	3.5153 (19)	H11D···H12C^ix^	2.3700
C2···H12B	2.6300	H11E···O2	2.5800
C4···H1^viii^	2.734 (17)	H11E···C11^x^	2.9400
C5···H12B^iv^	3.0400	H11F···C14^iii^	2.9100
C5···H1^viii^	2.782 (17)	H11F···H14B^iii^	2.3300
C6···H9B^iv^	2.8200	H12A···O1	2.8400
C6···H9B	3.0500	H12A···H4N	2.5400
C7···H9B^iv^	2.9100	H12B···C2	2.6300
C7···H9B	2.5400	H12B···H9B	2.4100
C9···H8^viii^	2.948 (17)	H12B···C5^viii^	3.0400
C9···H5^ii^	3.0800	H12C···N4	2.5500
C9···H6^viii^	3.0200	H12C···H4N	2.0800
C9···H2N	2.390 (19)	H12C···O2^vi^	2.7500
C10···H14A^iii^	2.9400	H12C···C11^vii^	2.8900
C11···H14B^iii^	3.0400	H12C···H11C^vii^	2.5100
C11···H12C^ix^	2.8900	H12C···H11D^vii^	2.3700
C11···H11E^x^	2.9400	H14A···O3	2.7200
C12···H11B^vii^	3.0700	H14A···O2^ii^	2.7100
C12···H11D^vii^	2.9700	H14A···C10^ii^	2.9400
C12···H4N	2.473 (19)	H14B···C11^ii^	3.0400
C13···H11D^vii^	2.8700	H14B···H11F^ii^	2.3300
C14···H11F^ii^	2.9100	H14C···O3	2.7100
H1···O3	2.263 (17)	H14C···O3^v^	2.6100
			
C1—O1—C8	110.78 (9)	C5—C6—H6	121.00
C8—N1—C9	118.52 (10)	C7—C6—H6	121.00
C8—N1—C10	119.14 (11)	O1—C8—H8	109.8 (10)
C9—N1—C10	121.77 (11)	N1—C8—H8	105.5 (10)
C10—N2—C11	121.32 (12)	C7—C8—H8	112.3 (10)
C1—N3—C12	117.44 (11)	N1—C9—H9A	109.00
C1—N3—C13	117.53 (11)	N1—C9—H9B	109.00
C12—N3—C13	122.57 (11)	N1—C9—H9C	109.00
C13—N4—C14	119.84 (12)	H9A—C9—H9B	109.00
C10—N2—H2N	120.3 (14)	H9A—C9—H9C	109.00
C11—N2—H2N	117.7 (14)	H9B—C9—H9C	109.00
C13—N4—H4N	124.3 (13)	N2—C11—H11A	109.00
C14—N4—H4N	115.9 (13)	N2—C11—H11B	109.00
O1—C1—N3	109.92 (10)	N2—C11—H11C	109.00
O1—C1—C2	104.04 (10)	N2—C11—H11D	109.00
N3—C1—C2	115.72 (11)	N2—C11—H11E	109.00
C1—C2—C7	109.48 (11)	N2—C11—H11F	109.00
C3—C2—C7	121.18 (12)	H11A—C11—H11B	109.00
C1—C2—C3	129.31 (12)	H11A—C11—H11C	109.00
C2—C3—C4	118.23 (13)	H11A—C11—H11D	141.00
C3—C4—C5	120.51 (13)	H11A—C11—H11E	56.00
C4—C5—C6	121.07 (13)	H11A—C11—H11F	56.00
C5—C6—C7	117.92 (13)	H11B—C11—H11C	109.00
C2—C7—C8	109.62 (11)	H11B—C11—H11D	56.00
C6—C7—C8	129.29 (12)	H11B—C11—H11E	141.00
C2—C7—C6	121.08 (12)	H11B—C11—H11F	56.00
O1—C8—N1	110.52 (11)	H11C—C11—H11D	56.00
O1—C8—C7	104.04 (10)	H11C—C11—H11E	56.00
N1—C8—C7	114.64 (11)	H11C—C11—H11F	141.00
O2—C10—N1	121.74 (12)	H11D—C11—H11E	109.00
O2—C10—N2	122.68 (12)	H11D—C11—H11F	109.00
N1—C10—N2	115.53 (12)	H11E—C11—H11F	109.00
O3—C13—N4	122.04 (12)	N3—C12—H12A	109.00
N3—C13—N4	116.78 (11)	N3—C12—H12B	109.00
O3—C13—N3	121.16 (12)	N3—C12—H12C	109.00
O1—C1—H1	109.5 (10)	H12A—C12—H12B	109.00
N3—C1—H1	109.2 (10)	H12A—C12—H12C	109.00
C2—C1—H1	108.2 (10)	H12B—C12—H12C	109.00
C2—C3—H3	121.00	N4—C14—H14A	109.00
C4—C3—H3	121.00	N4—C14—H14B	109.00
C3—C4—H4	120.00	N4—C14—H14C	109.00
C5—C4—H4	120.00	H14A—C14—H14B	109.00
C4—C5—H5	119.00	H14A—C14—H14C	109.00
C6—C5—H5	119.00	H14B—C14—H14C	109.00
			
C8—O1—C1—N3	−138.76 (11)	C14—N4—C13—O3	−6.6 (2)
C8—O1—C1—C2	−14.26 (13)	C14—N4—C13—N3	171.61 (12)
C1—O1—C8—N1	137.61 (11)	O1—C1—C2—C3	−173.35 (13)
C1—O1—C8—C7	14.08 (13)	O1—C1—C2—C7	8.71 (14)
C9—N1—C8—O1	−66.82 (14)	N3—C1—C2—C3	−52.67 (19)
C9—N1—C8—C7	50.35 (16)	N3—C1—C2—C7	129.39 (12)
C10—N1—C8—O1	104.63 (13)	C1—C2—C3—C4	−178.04 (13)
C10—N1—C8—C7	−138.20 (12)	C7—C2—C3—C4	−0.3 (2)
C8—N1—C10—O2	13.95 (19)	C1—C2—C7—C6	178.74 (12)
C8—N1—C10—N2	−168.36 (12)	C1—C2—C7—C8	−0.30 (15)
C9—N1—C10—O2	−174.89 (12)	C3—C2—C7—C6	0.6 (2)
C9—N1—C10—N2	2.80 (18)	C3—C2—C7—C8	−178.44 (12)
C11—N2—C10—O2	1.7 (2)	C2—C3—C4—C5	0.4 (2)
C11—N2—C10—N1	−176.00 (12)	C3—C4—C5—C6	−0.8 (2)
C12—N3—C1—O1	58.72 (15)	C4—C5—C6—C7	1.0 (2)
C12—N3—C1—C2	−58.73 (15)	C5—C6—C7—C2	−0.9 (2)
C13—N3—C1—O1	−103.94 (13)	C5—C6—C7—C8	177.90 (13)
C13—N3—C1—C2	138.62 (12)	C2—C7—C8—O1	−8.26 (14)
C1—N3—C13—O3	−17.51 (18)	C2—C7—C8—N1	−129.07 (12)
C1—N3—C13—N4	164.25 (12)	C6—C7—C8—O1	172.81 (13)
C12—N3—C13—O3	−179.21 (12)	C6—C7—C8—N1	51.99 (19)
C12—N3—C13—N4	2.54 (18)		

Symmetry codes: (i) −*x*+1, *y*+1/2, −*z*+1/2; (ii) *x*+1/2, *y*, −*z*+1/2; (iii) *x*−1/2, *y*, −*z*+1/2; (iv) −*x*+1/2, *y*+1/2, *z*; (v) −*x*+1, −*y*+1, −*z*; (vi) −*x*+1, *y*−1/2, −*z*+1/2; (vii) *x*, −*y*+1/2, *z*−1/2; (viii) −*x*+1/2, *y*−1/2, *z*; (ix) *x*, −*y*+1/2, *z*+1/2; (x) −*x*+1, −*y*+1, −*z*+1.

### Hydrogen-bond geometry (Å, °)

**Table:**

*D*—H···*A*	*D*—H	H···*A*	*D*···*A*	*D*—H···*A*
N2—H2N···O3^vi^	0.806 (19)	2.062 (19)	2.8229 (16)	157.4 (19)
N4—H4N···O2^vi^	0.858 (18)	2.006 (18)	2.8322 (15)	161.2 (18)
C1—H1···O3	0.987 (17)	2.263 (17)	2.7205 (16)	107.0 (12)
C8—H8···O2	1.003 (17)	2.239 (17)	2.7505 (17)	110.1 (12)
C11—H11A···O2	0.96	2.39	2.7730 (18)	103.0
C9—H9B···CgA	0.96	2.6600	3.0207 (14)	103.0
C12—H12B···CgA	0.96	2.7200	3.0046 (15)	98.0

Symmetry codes: (vi) −*x*+1, *y*−1/2, −*z*+1/2.
